# Purine metabolism controls innate lymphoid cell function and protects against intestinal injury

**DOI:** 10.1111/imcb.12167

**Published:** 2018-07-10

**Authors:** Siobhan Crittenden, Ashleigh Cheyne, Alexander Adams, Thorsten Forster, Calum T Robb, Jennifer Felton, Gwo‐Tzer Ho, Dominik Ruckerl, Adriano G Rossi, Stephen M Anderton, Peter Ghazal, Jack Satsangi, Sarah E Howie, Chengcan Yao

**Affiliations:** ^1^ Medical Research Council (MRC) Centre for Inflammation Research Queen's Medical Research Institute The University of Edinburgh Edinburgh EH16 4TJ UK; ^2^ Gastrointestinal Unit Institute of Genetics and Molecular Medicine Western General Hospital The University of Edinburgh Edinburgh EH4 2XU UK; ^3^ Division of Pathway Medicine Edinburgh Infectious Diseases The University of Edinburgh Edinburgh EH16 4SB UK; ^4^ Faculty of Biology, Medicine and Health School of Biological Sciences The University of Manchester Manchester M13 9PT UK; ^5^ Centre for Synthetic and Systems Biology (SynthSys) The University of Edinburgh Edinburgh EH9 3JD UK

**Keywords:** Adenosine, ectonucleotidase (NTPDase), IL‐22, intestinal injury, purinergic signaling, type 3 innate lymphoid cell (ILC3)

## Abstract

Inflammatory bowel disease (IBD) is a condition of chronic inflammatory intestinal disorder with increasing prevalence but limited effective therapies. The purine metabolic pathway is involved in various inflammatory processes including IBD. However, the mechanisms through which purine metabolism modulates IBD remain to be established. Here, we found that mucosal expression of genes involved in the purine metabolic pathway is altered in patients with active ulcerative colitis (UC), which is associated with elevated gene expression signatures of the group 3 innate lymphoid cell (ILC3)–interleukin (IL)‐22 pathway. In mice, blockade of ectonucleotidases (NTPDases), critical enzymes for purine metabolism by hydrolysis of extracellular adenosine 5′‐triphosphate (eATP) into adenosine, exacerbates dextran‐sulfate sodium‐induced intestinal injury. This exacerbation of colitis is associated with reduction of colonic IL‐22‐producing ILC3s, which afford essential protection against intestinal inflammation, and is rescued by exogenous IL‐22. Mechanistically, activation of ILC3s for IL‐22 production is reciprocally mediated by eATP and adenosine. These findings reveal that the NTPDase‐mediated balance between eATP and adenosine regulates ILC3 cell function to provide protection against intestinal injury and suggest potential therapeutic strategies for treating IBD by targeting the purine–ILC3 axis.

## Introduction

The purinergic signaling pathway plays essential roles in physiological and pathophysiological processes in a variety of organs. An incompetent purine metabolic pathway has been identified to be associated with excessive inflammation and inappropriate resolution in numerous human inflammatory diseases, including inflammatory bowel disease (IBD), ischemia, diabetes and cancer.^1^, ^2^, ^3^ Extracellular nucleotides such as extracellular adenosine 5′‐triphosphate (eATP) that are released during tissue damage can drive intestinal inflammation by acting as a damage‐associated molecular pattern.^4^, ^5^ Cell‐surface ectonucleotidases (NTPDases) such as NTPDase1 (i.e. CD39), together with the ecto‐5′‐nucleotidase CD73, hydrolyze eATP to adenosine, a purine nucleoside with anti‐inflammatory effects.^3^, ^6^, ^7^ NTPDase1 is present at high levels in intestinal tissues from patients with IBD.^8^ Furthermore, gene polymorphisms of *ENTPD1* link to the human immune system under homeostatic and inflammatory conditions (e.g. IBD).^9^, ^10^ These lines of evidence suggest that the control of purine metabolism is important in IBD.

Recent studies have highlighted that the metabolism of purine nucleotides by NTPDases has a direct role in modulation of regulatory T cells (Tregs) and interleukin (IL)‐17‐producing Th17 cell functions,^11^, ^12^, ^13^ both important in the genesis of pathogenic T‐cell responses that form the characteristic of IBD.^14^ Interestingly, a further subset of immune cells, group 3 innate lymphoid cells (ILC3s), has also been shown to play a major role in gut mucosal immunity and inflammation.^15^, ^16^, ^17^ ILC3s are a group of lymphocytes without T‐cell‐specific antigen receptors with particular accumulation at mucosal sites and serve as key regulators during intestinal inflammation.^18^, ^19^, ^20^, ^21^ ILC3s can rapidly respond to external stimuli and play critical roles in host defense, the resolution of inflammation and initiation of tissue repair in the gut.^21^, ^22^, ^23^, ^24^, ^25^, ^26^ These protective effects of ILC3s are mostly dependent on their production of the reparative cytokine IL‐22. By binding to its receptor on epithelial cells, IL‐22 contributes to production of antimicrobial peptides and mucins, regulation of gut microbiota, maintenance of the gut barrier function and amelioration of intestinal inflammation.^25^, ^26^ But it remains unclear how ILC3s are activated and regulated during intestinal inflammation.

Here, we have explored whether the purine pathway signals modulate ILC3 function and whether this is involved in regulation of intestinal injury in the animal model. We have also investigated whether alterations in purine metabolism are associated with activation of the ILC3‐IL‐22 axis in human inflamed colon biopsies by analyzing expression profiles. Our results demonstrate that targeting purine metabolism and innate lymphocytes would benefit developing new therapies against inflammatory diseases such as IBD.

## Results

### Alterations in purine metabolism correlate with the ILC3/Th17‐IL‐22 pathway in human inflamed colon

**Figure 1:**
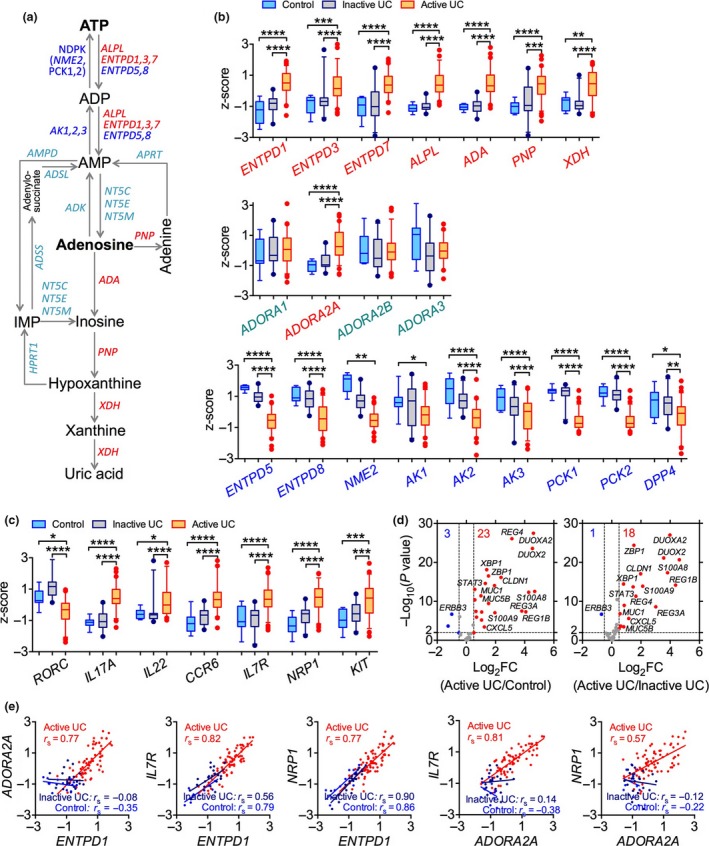
Alterations in purine metabolism and IL‐22 response in patients with intestinal inflammation. **(a)** Schematic depicting the purine metabolic pathway and enzymes involved. **(b)** Expression of enzymes involved in purine metabolism and adenosine receptors in colonic mucosal biopsies of patients suffering from active ulcerative colitis (UC) (*n* = 72), inactive UC (*n* = 23) or controls with normal mucosa (*n* = 11) as described.^27^ **(c)** Expression of selected human ILC3 and Th17 related genes. **(d)** Volcano plot for changes in expression of IL‐22 signaling‐responsive genes in colonic mucosal biopsies with active UC as compared to those in colonic mucosal biopsies from normal controls or with inactive UC. Each point represents one single probe. Genes were considered biologically relevant and statistically significant with fold‐change (FC) ≥ 1.4 and *P *≤* *0.01, respectively. **(e)** Correlations between *ADORA2A* with *ENTPD1* expression and their correlations with ILC3 genes *IL7R* and *NRP1* in colonic biopsy samples from patients with active or inactive UC or controls. Microarray data were retrieved from the Gene Expression Ominibus dataset GSE59071^27^ and the z‐score transformed values (by gene across samples) of microarray gene expression data are shown as box and whiskers with 5–95 percentiles. *P <* *0.05; **P <* *0.01; ***P <* *0.001; ****P <* *0.0001 by a nonparametric Kruskal–Wallis test with *post hoc* Dunn's multiple comparisons test **(b, c)**. A nonparametric Spearman correlation test was performed and each point represents an individual subject **(e)**.

We analyzed the gene expression profiles of whole colonic mucosal biopsies from patients with active or inactive ulcerative colitis (UC) and control individuals by re‐analyzing a public microarray dataset,^27^ with a particular interest in the purine metabolic pathway (Figure 1a). We found that expression of genes related to the ATP degradation pathway was altered in inflamed colons compared to unaffected areas of the colon and in colons of individuals without IBD. For example, NTPDase genes (e.g. *ENTPD1,3,7)* were upregulated in active UC colons compared to inactive UC or control colons (Figure 1b), while adenylate kinase genes (e.g. *AK1‐3*) and nucleoside‐diphosphate kinase genes (NDPK, e.g. *NME2, PCK1/2*) that catalyze the interconversion of adenine nucleotides to ATP were downregulated in active UC colon biopsies compared to inactive or normal control colon biopsies (Figure 1b). While genes *PNP* and *XDH* were differentially expressed in active UC colon, expression of 5′‐nucleotidase genes (e.g. *NT5E*) and other ATP metabolism related genes (e.g. *AMPD, APRT, ADK, ADSS, ADSL*) were not changed among colons with different states (Figure 1b and data not shown). Although adenosine deaminase gene expression was upregulated, expression of *DPP4* gene, encoding the dipeptidylpeptidase IV (CD26) that directly interacts with and controls adenosine deaminase activity,^28^ was downregulated in active UC colons (Figure 1b), suggesting that active UC colons unlikely display effective deamination of adenosine. Moreover, expression of *ADORA2A* (encoding adenosine receptor A2A), rather than other adenosine receptors, is upregulated in actively inflamed colon biopsies (Figure 1). These results indicate that there may be downregulated ATP levels but enhanced adenosine signaling, probably through the A2A receptor, in inflamed colons.

We also observed that expression of ILC3 and Th17 genes^29^, ^30^, ^31^ distinguished active UC from inactive UC and controls although there were a few patient‐to‐patient variations in the gene expression levels within the active UC group (Supplementary figures 1, 2). For example, human ILC3 genes (e.g. *IL7R, NRP1, KIT*), Th17 genes (e.g. *IL23A, IL17A, IL17F*) and ILC3/Th17 co‐expressing genes (e.g. *CCR6* and *IL22*) were upregulated in colon biopsies from active UC patients compared to controls (Figure 1c). Importantly, inflamed colons developed a strong IL‐22 response^32^ marked by the overexpression of genes related to activation of IL‐22 receptor signaling (e.g. *STAT3, REG3A, S100A8, MUC1, CXCL5*) in active UC colons compared to inactive UC or control colons (Figure 1d). These results are consistent with a previous study showing that IBD patients have more IL‐22‐producing ILC3s in colonic mucosa than control individuals.^33^ Interestingly, in the mucosal tissue of inflamed colons there was a positive correlation between expression of *ENTPD1* and *ADORA2A* genes, and both genes positively correlated with ILC3‐signature genes (e.g. *IL7R* and *NRP1*) in active UC colon biopsies (Figure 1e). To determine whether these findings are unique to this population we investigated another independent cohort.^34^ In this independent investigation we also found similar pattern of expression, particularly, the overexpression of *ENTPD1* and *ADORA2A* and alterations in expression of the ILC3‐IL‐22 axis‐related and purine metabolic pathway genes in colon biopsies from UC patients (Supplementary figure3). These findings collectively support a potential interaction between the purine metabolic pathway, especially the ATP/NTPDase/adenosine signaling pathway, and the ILC3‐IL‐22 axis in the setting of intestinal inflammation. Given that both the purinergic and ILC3‐IL‐22 pathways have been indicated to regulate intestinal inflammation, these genetic results prompted us to investigate whether the purinergic signaling has a role in modulation of ILC3‐IL‐22 pathway in response to gut epithelial injury.

### NTPDase inhibition results in more severe intestinal injury and reduction of mucosal ILC3s

**Figure 2:**
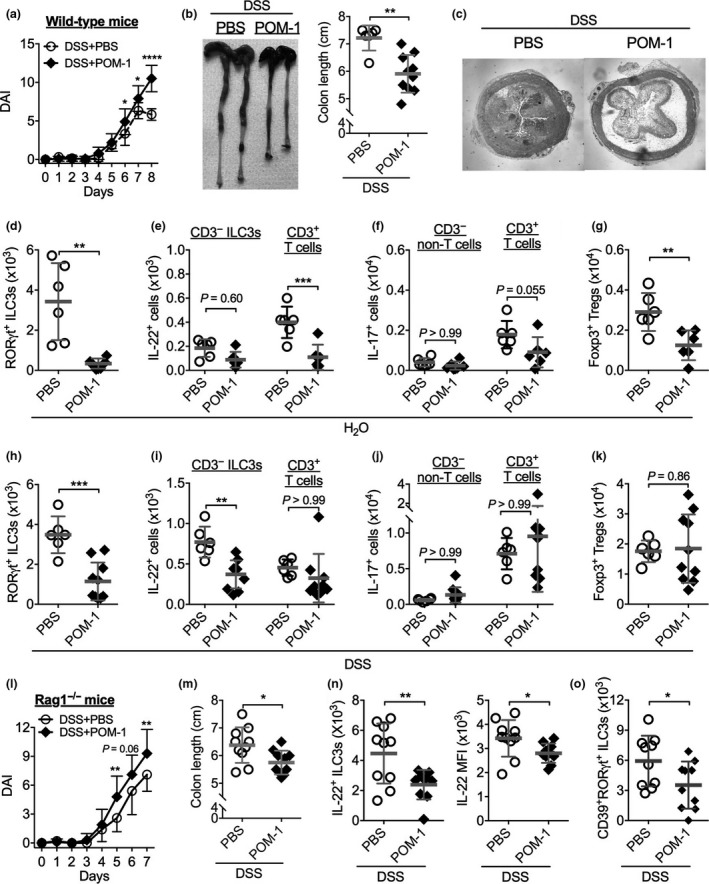
Inhibition of NTPDases exacerbates dextran‐sulphate sodium (DSS)‐induced intestinal damage, which is associated with reduction of IL‐22‐producing ILC3s. Wild‐type C57BL/6 mice were treated with 1.5% DSS **(a–c, h–k)** or not **(d**–**g)** in drinking water from Day 0 to Day 6 and intreperitoneally (i.p.) injected with an NTPDase inhibitor POM‐1 or PBS from Day 2 to Day 7 daily. **(a)** Disease activity index (DAI). **(b)** Representative colons and colon length. **(c)** Representative H&E–stained images of mouse proximal colon samples. **(d–g)** Numbers of colonic lamina propria CD45^+^Lin^−^ CD90.2^+^ RORγt^+^ ILC3s **(d)**, IL‐22^+^ ILC3s and T cells **(e)**, IL‐17^+^ CD3^+^ T and IL‐17^+^ CD3^−^ non‐T cells **(f)**, and Foxp3^+^ CD3^+^ Tregs **(g)** from mice treated with PBS (*n* = 6) or POM‐1 (*n* = 6) detected by flow cytometry. **(h**–**k)** Numbers of colonic lamina propria CD45^+^Lin^−^ CD90.2^+^ RORγt^+^ ILC3s **(h)**, IL‐22^+^ ILC3s and T cells **(i)**, IL‐17^+^ CD3^+^ T and IL‐17^+^ CD3^−^ non‐T cells **(j)**, and Foxp3^+^ CD3^+^ Tregs **(k)** from mice treated with dextran‐sulphate sodium (DSS) plus PBS (*n* = 6) or POM‐1 (*n* = 9) detected by flow cytometry. **(l–o)** *Rag1* ^−*/*−^ mice were treated with 1.5% DSS in drinking water from Day 0 to Day 5. POM‐1 (*n* = 10) or PBS (*n* = 10) was injected into mice from Day 2 to Day 6 daily. **(l)** Disease activity index (DAI). **(m)** Colon length. **(n)** Numbers of colonic LP IL‐22^+^ ILC3s and mean fluorescent intensity (MFI) of IL‐22. **(o)** Numbers of colonic LP CD39^+^ RORγt^+^ ILC3s. Data shown are mean ±  s.d. pooled from two independent experiments. Each point represents one individual mouse. *P <* *0.05; **P <* *0.01; ***P <* *0.001 by two‐way ANOVA with a *post hoc* Bonferroni's multiple comparisons test **(a, l)** or a two‐tailed unpaired Student's *t*‐test **(b, d**–**k, m–o)**.

To address the potential interaction between the NTPDase‐mediated purine metabolic pathway and the ILC3‐IL‐22 axis and its role in intestinal injury, we used the dextran‐sulfate sodium (DSS)‐induced intestinal injury model and pharmacologically inhibited the function of NTPDases using a small molecule compound sodium polyoxotungstate 1 (POM‐1). POM‐1 inhibits phosphohydrolysis of ATP by selectively targeting NTPDases, especially NTPDase1 and NTPDase3, both overexpressed in human inflamed intestinal mucosa (Figure 1b).^35^ It has been reported that global deficiency of NTPDase1 or NTPDase3 resulted in exacerbation of DSS colitis.^9^, ^36^ DSS administration induced similar body weight loss in both control and POM‐1‐treated mice. However, POM‐1 treatment led to more severe intestinal bleeding and reduced mice general appearance, leading to augmented colonic inflammation, evidenced by shortened colon and more severe pathology compared to control mice (Figure 2a–c). Mice treated with POM‐1 in the absence of DSS did not develop any sign of intestinal inflammation (data not shown). Our results are in agreement with a previous report showing that administration of POM‐1 enhanced extracellular ATP levels in colons and worsened DSS‐induced colitis while administration of apyrase, an enzyme mediating ATP degradation, ameliorates DSS‐colitis.^37^ To gain insight into the mechanisms driving NTPDase‐dependent attenuation of colonic damage, we analyzed adaptive and innate lymphocytes in the colon by flow cytometry. Previous studies have shown that epithelial damage (e.g. induced by DSS) led to expansion and activation of RAR‐related orphan receptor gamma t (RORγt)^+^ ILC3s, and that deactivation of ILC3s reduced IL‐22 production and delayed the recovery from colitis.^20^ We thus investigated the relationship between NTPDase inhibition and intestinal ILC3s in the steady state and during colonic inflammation. We found that in steady state, POM‐1 treatment significantly reduced accumulation of CD45^+^Lineage (Lin, CD3/B220/CD11c/CD11b/NK1.1)^−^CD90.2^+^RORγt^+^ ILC3s, but not activated IL‐22‐producing ILC3s, in the colon (Figure 2d, e and Supplementary figure 4). In addition, POM‐1 also decreased colonic IL‐22^+^CD3^+^ T cells as well as Foxp3^+^ Tregs (Figure 2e–g), the latter was in agreement with previous findings showing that NTPDases (especially CD39) are critical for Treg development and function.^11^ Administration of DSS did not change the numbers of RORγt^+^ ILC3s in the colon but increased colonic IL‐22^+^ ILC3s by ~4‐fold while colonic IL‐22^+^CD3^+^ T cells were not changed by DSS (Figure 2d, e, h, i). Co‐administration of POM‐1 significantly prevented DSS‐dependent increase in IL‐22^+^ ILC3s but did not affect any T‐cell subsets examined compared to that in mice treated with DSS and vehicle control (Figure 2h–k), suggesting that ILC3s are the main possible source of IL‐22 produced in response to acute gut damage and they are negatively correlated with intestinal damage. DSS alone also increased accumulation of colonic IL‐17^+^CD3^+^ T cells and Foxp3^+^ Tregs, but these T‐cell subsets were not affected further by POM‐1 co‐treatment (Figure 2f, g, j, k). These data indicate that NTPDase‐dependent protection against acute intestinal injury is likely associated with IL‐22‐producing ILC3s.

To further study the involvement of non‐T cells in control of gut inflammation by NTPDases, we used the DSS‐induced colonic injury model in *Rag1* ^−*/*−^ mice that have no T and B cells. Similar to WT C57BL/6 mice, POM‐1 treatment increased the severity of DSS‐induced colitis in *Rag1* ^−*/*−^ mice and shortened colon length (Figure 2l, m). In these mice, POM‐1 treatment again reduced both the frequency and absolute number of IL‐22^+^ ILC3s in the colon (Figure 2n). Furthermore, POM‐1 treatment decreased IL‐22 mean fluorescent intensity (MFI) in ILC3s, suggesting an inhibitory effect of POM‐1 on IL‐22 production from ILC3s at the single‐cell level (Figure 2n). Moreover, POM‐1 treatment also decreased colonic ILC3 expression of CD39, the key ENTPDase encoded by *ENTPD1* gene (Figure 2o). These results, together with results of the downregulation of RORγt and IL‐22 expression in ILC3s, further confirm our findings about gene expression of the purinergic and ILC3/IL‐22 pathways in human colon biopsies.

### ILC3 produced IL‐22 mediates NTPDase control of intestinal inflammation

**Figure 3:**
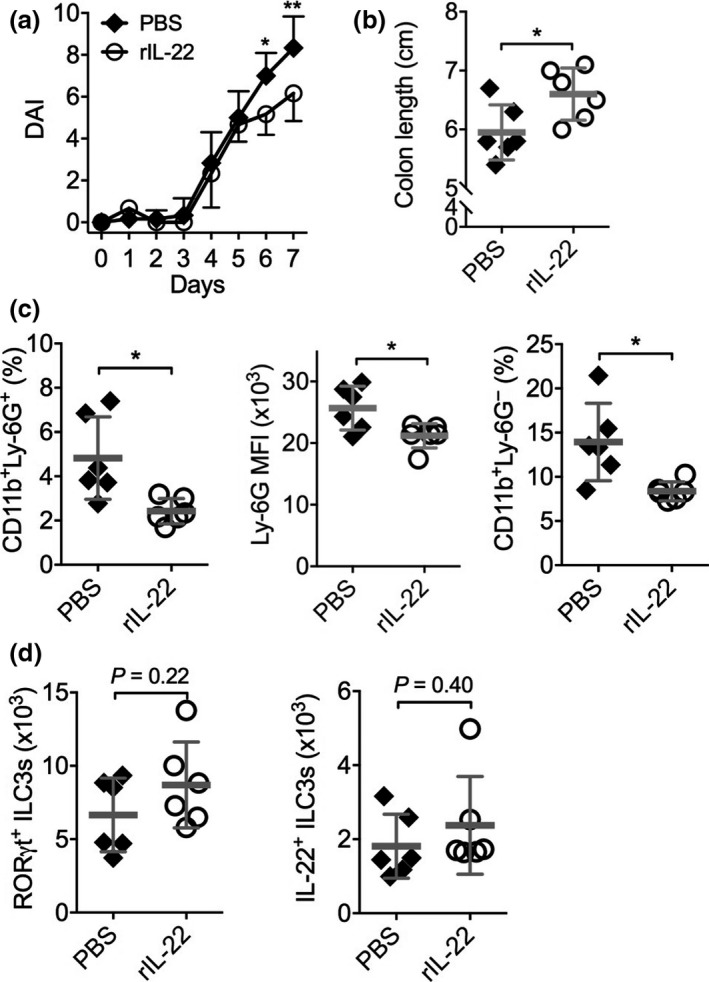
Exacerbation of intestinal damage by NTPDase inhibition is rescued by exogenous IL‐22. *Rag1* ^−*/*−^ mice were treated with 1.5% dextran‐sulfate sodium (DSS) in drinking water from Day 0 to Day 5. POM‐1 was injected from Day 2 to Day 6 daily, and rIL‐22 (*n* = 6) or PBS (*n* = 6) was injected from Day 0 to Day 6 daily. **(a)** DAI. **(b)** Colon length. **(c)** Numbers of colonic LP CD11b^+^Ly‐6G^+^ neutrophils (left), Ly‐6G MFI (middle) and CD11b^+^Ly‐6G^−^ macrophages (right) determined by flow cytometry. **(d)** Numbers of colonic LP CD45^+^Lin^−^ CD90.2^+^ RORγt^+^ ILC3s (left) and IL‐22^+^ ILC3s (right). Data shown are mean ± s.d. from one experiment. Each point represents one individual mouse. *P <* *0.05; **P <* *0.01 by two‐way ANOVA with a *post hoc* Bonferroni's multiple comparisons test **(a)** or a two‐tailed unpaired Student's *t*‐test **(b**‐**d)**. NS, not significant.

Because IL‐22 is critical for protection against intestinal barrier damage and acceleration of the epithelial repair post injury.^22^, ^23^, ^24^, ^25^, ^26^ We thus asked whether exacerbation of colonic damage by NTPDase inhibition is a consequence of the reduction of IL‐22 production from colonic ILC3s. To address this question, we treated *Rag1* ^−*/*−^ mice with DSS and POM‐1, and a subgroup of mice were administrated with recombinant mouse IL‐22 (rIL‐22). Indeed, administration of rIL‐22 ameliorated POM‐1‐dependent augmentation of colitis disease activity (Figure 3a), reversed the shortening of colon length (Figure 3b) and reduced the infiltration of CD11b^+^Ly‐6G^+^ neutrophils and CD11b^+^Ly‐6G^−^ macrophages in the colon (Figure 3c). However, neither RORγt^+^ ILC3s nor endogenous IL‐22 production in the colon were affected by rIL‐22 (Figure 3d). These results suggest that ILC3‐producing IL‐22 is important for control of colitis.

### ATP and adenosine reciprocally regulate IL‐22 production from ILC3s *in vitro*

**Figure 4:**
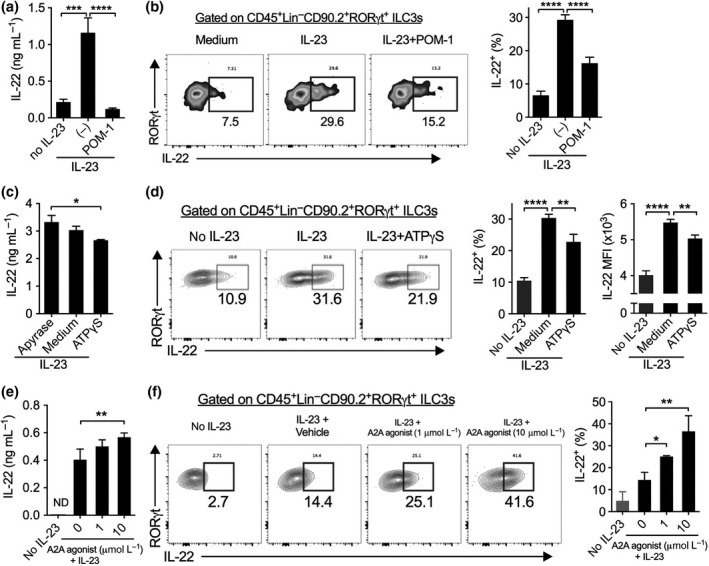
ATP and adenosine reciprocally regulate IL‐22 production from ILC3s *in vitro*. **(a, b)** Concentrations of IL‐22 in supernatants **(a)** and percentages of IL‐22^+^ ILC3s **(b)** of small intestinal lamina propria leukocytes (siLPLs) isolated from *Rag1* ^−*/*−^ mice and cultured without or with IL‐23 plus POM‐1 or not overnight **(a)** or for 4 h **(b)**. **(c, d)** Concentrations of IL‐22 in supernatants **(c)** and percentages of IL‐22^+^ ILC3s **(d)** of LP leukocytes isolated from small intestine of *Rag1* ^−*/*−^ mice and then cultured with IL‐23 plus Apyrase or ATPγS overnight **(c)** or for 4 h **(d)**. **(e, f)** Concentrations of IL‐22 in supernatants **(e)** and percentages of IL‐22^+^ ILC3s **(f)** of spleen cells isolated from *Rag1* ^−*/*−^ mice and then cultured with IL‐23 plus indicated concentrations of an A2A receptor agonist CGS21680 overnight **(e)** or for 4 h **(f)**. Numbers adjacent to outlined areas indicate percent RORγt^+^ IL‐22^+^ ILC3s. Data shown are mean ± s.d., representative from two or more independent experiments. *P <* *0.05, **P <* *0.01, ***P <* *0.001; ****P <* *0.0001 by one‐way ANOVA with a *post hoc* Bonferroni's multiple comparisons test.

To investigate how NTPDase influences ILC3 activation, we isolated small intestinal lamina propria leukocytes from *Rag1* ^−*/*−^ mice and cultured them with IL‐23 *in vitro*. IL‐23 induced IL‐22 production from lamina propria leukocytes, which was almost completely prevented by POM‐1 (Figure 4a). Flow cytometric analysis confirmed that IL‐22 was produced by CD45^+^Lin^−^CD90.2^+^RORγt^+^ ILC3s in response to IL‐23 stimulation, and IL‐22 production from ILC3s were reduced by POM‐1 (Figure 4b). Similarly, POM‐1 also reduced IL‐22 production from spleen ILC3s (Supplementary figure 5a, b).

Inhibition of NTPDases prevents eATP hydrolysis, leading to increase in eATP levels and reduction of its metabolites such as adenosine.^6^, ^7^ We further investigated the effects of eATP and adenosine on IL‐22 production from ILC3s. We used apyrase (which mimics NTPDases to mediate ATP hydrolysis) and ATPγS (a non‐hydrolyzed ATP analogue) to modulate the levels of eATP. Increasing eATP levels (e.g. by addition of ATPγS) diminished IL‐22 production from ILC3s, while reducing eATP levels (e.g. through apyrase‐inducing ATP breakdown) increased IL‐22 production by either intestinal or splenic ILC3s (Figure 4c, d and Supplementary figure 5c, d). Reducing the levels of eATP also increased IL‐22 MFI among IL‐22^+^ ILC3s (Figure 4d). In contrast, activation of adenosine receptor A2A by a selective agonist CGS21680 increased ILC3 production of IL‐22 in a concentration‐dependent manner (Figure 4e, f). This is in agreement with our observation that ILC3s highly express the A2A gene (encoded by *Adora2a*), rather than other adenosine receptors (Supplementary figure 6). These results indicate that the balance of eATP and adenosine, which is controlled by NTPDases, reciprocally regulates IL‐22 production from ILC3s.

## Discussion

**Figure 5:**
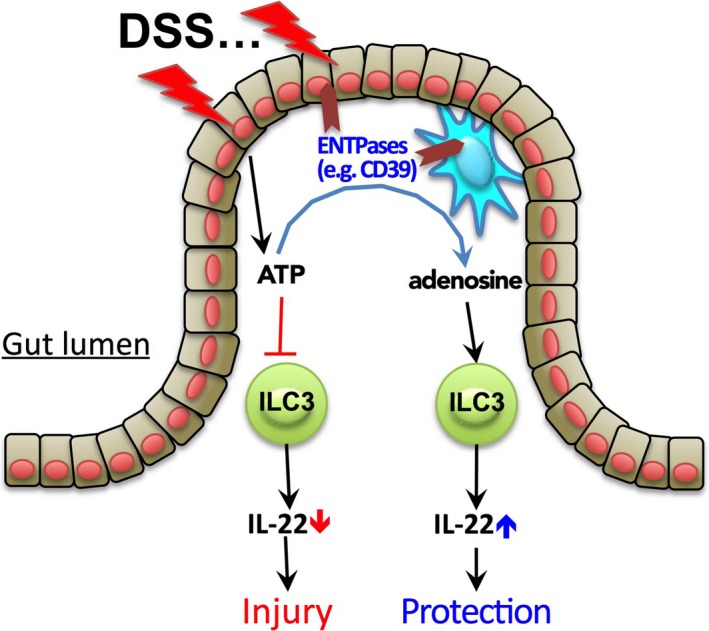
A proposed model for how the ATP/NTPDase/adenosine axis regulates ILC3s during intestinal injury. In response to dextran‐sulphate sodium (DSS) stimulation, injured epithelial cells produce a large amount of extracellular ATP, which targets on ILC3s by inhibiting IL‐22 production, leading to exacerbation of intestinal barrier damage. Simultaneously, tissue damage also induces expression of NTPDases (e.g. CD39 and CD73) by many types of cells (e.g. epithelial cells, mononuclear phagocytes and T cells). NTPDases in turn convert extracellular ATP into adenosine. Unlike ATP, however, adenosine promotes IL‐22 production and ILC3 activation through its receptor A2A, and therefore serves to protect against intestinal damage. NTPDases thus regulate ILC3 activation and gut injury by modulating the balance of extracellular ATP and adenosine.

Here, we demonstrate that NTPDases, the key enzymes in the purine metabolic pathway, have a crucial role in protection against intestinal injury through controlling the balance between ATP and its metabolite adenosine that reciprocally regulate ILC3 activation (Figure 5). These findings are consistent with previous reports that ATP and adenosine exacerbates and protects against gut inflammation, respectively.^37^, ^38^, ^39^, ^40^ Mice deficient in NTPDases have more severe DSS‐induced colitis, and human *ENTPD1* gene polymorphisms increased IBD susceptibility and also affected the homeostasis of the immune system.^9^, ^36^ Our results indicate that these protective roles of NTPDases are also likely to be mediated through modulating innate immune responses (e.g. ILC3s) in addition to reported mechanisms via regulation of the adaptive immune system (e.g. Treg and Th17/Th1 cells).^11^, ^12^, ^13^

At the early stage of mucosal injury, damaged epithelial cells and activated immune cells release ATP into the microenvironment of mucosal tissue. As a damage‐associated molecular pattern, eATP can also trigger the activation of the NLRP3 inflammasome and induce IL‐8 and IL‐1β production.^41^ Besides this effect, our work indicates that increase in the levels of eATP can also prevent ILC3 activation and subsequent IL‐22 production. ILC3s are the innate counterpart of Th17 cells, and both types of cells share the lineage‐determining transcription factors (e.g. RORγt), cytokine production profiles (e.g. IL‐22, IL‐17) and surface markers (e.g. IL‐23R, IL‐1R, CCR6).^18^ ATP has been shown to promote Th17 cell development through indirect actions on antigen‐presenting cells,^4^ but our findings suggest that ATP can also directly act on ILC3s to suppress their function and IL‐22 production.

Injury of the gut epithelium, e.g. induced by DSS, not only increases colonic eATP levels^37^ but also upregulates NTPDases such as CD39.^42^ During the course of mucosal inflammation, increasing expression of NTPDases leads to ATP breakdown and release of extracellular adenosine, which has been implicated as an endogenous protective reagent in IBD.^43^ A2A and A2B receptors were suggested to contribute to the anti‐inflammatory action of adenosine.^44^, ^45^, ^46^ We found here that ILC3s express high levels of A2A receptor, and activation of the A2A receptor promotes IL‐22 production from ILC3s. Consistently, gene expression of A2A rather than other adenosine receptors was upregulated in inflamed colons compared to control colons in humans. As the A2A receptor dominantly activates the cAMP signaling pathway,^7^ these results are consistent with our previous findings that ILC3 activation and IL‐22 production were positively regulated by another cAMP elevating reagent prostaglandin E_2._ 22 Therefore, both downregulation of ATP signaling and upregulation of adenosine signaling may contribute to NTPDase‐dependent enhancing IL‐22 production from ILC3s, leading to protection against intestinal inflammation.

In agreement with our findings that the ATP/NTPDase/adenosine pathway modulates ILC3 activation and acute colonic inflammation in mice, microarray analysis showed striking alterations in gene expression of the purine metabolic pathway in the inflamed colon from patients with active UC. While downregulation of nucleoside‐diphosphate kinases (e.g. *NME2, PCK1, PCK2*) and adenylate kinase (e.g. *AK1, AK2, AK3*) suggests a debilitated ATP biosynthetic process, upregulated expression of *ALPL* and NTPDases, especially *ENTPD1*, indicates the acceleration of ATP/ADP hydrolysis. These gene expression data suggest a possibility of reduction of the levels of eATP and increase of the levels of extracellular adenosine at the inflamed sites, together contributing to resolution of inflammation. Indeed, ATP levels were reduced in the intestine of patients with IBD,^47^, ^48^, ^49^ and expression of the A2A receptor was upregulated in inflamed colons. Studies have shown that selective agonists for activation of the A2A receptor reduced tissue damage in various animal models of IBD.^44^, ^50^ Our results provide new insights into how alterations in purine metabolism in response to tissue damage modulate the pathophysiology of IBD, that is, through interaction with the ILC3‐IL‐22 pathway. Given that drugs targeting the purinergic signaling for gastrointestinal diseases are being developed and accelerated toward testing in clinical trials,^51^, ^52^ our findings will help to clarify their therapeutic mechanisms.

Besides ILC3s, there are many other intestinal cells that also express NTPDases including CD39 and CD73. Because of the relatively rare cell number of ILC3s when compared to other types of cells (e.g. epithelial cells, mononuclear phagocytes and T lymphocytes) in the gut, it is unlikely that CD39^+^ ILC3s are the main cells responsible to NTPDase‐mediated regulation of eATP breakdown into adenosine. Therefore, IL‐22 production from ILC3s is more likely controlled by NTPDase expressed from other cells (e.g. epithelial cells and mononuclear phagocytes), rather than ILC3s themselves, in the gut. Further studies are required to elucidate the effects of NTPDase‐mediated eATP hydrolysis on ILC3 function and modulation of IBD using cell type‐dependent CD39 conditional knockout animals. Even though, the inverse correlation of colonic CD39^+^ ILC3 numbers and the intestinal inflammation severity indicates that CD39 expression on ILC3s may be potentially used as a biomarker for the IBD severity. Taken together, our work demonstrates that the balance between ATP and adenosine controlled by NTPDases regulates ILC3 cell behavior in the context of gut inflammation, suggesting that a combinational approach of using pharmacological inhibition of the ATP signaling, acceleration of extracellular conversion of ATP to adenosine and activation of the adenosine receptors represents a potential approach to improve the development of new therapeutic strategies against IBD.

## Methods

### Mice

Wild‐type C57BL/6 mice were purchased from Harlan UK. C57BL/6 *Rag1* ^−*/*−^ mice were bred and maintained under specific pathogen‐free conditions in accredited animal facilities at the University of Edinburgh. Mice used in this study were sex‐ and age (8–12 weeks old)‐matched. Mice were analyzed individually and no mice were excluded from the analysis, with the exception of exclusions due to technical errors in preparation of intestinal lamina propria leukocytes. All animal experiments were conducted in accordance with the UK Scientific Procedures Act of 1986 and approved by the local ethical approval.

### DSS colitis model

Treatment of DSS was performed on WT C57BL/6 or *Rag1* ^−*/*−^ mice.^22^ Briefly, WT C57BL/6 or *Rag1* ^−*/*−^ mice (8–9 weeks old at the beginning of experiments) were given 1.5% (w/v) of dextran sulfate sodium (DSS, MW 36–50 kDa, MP Biochemical) in drinking water for 6 or 5 consecutive days, respectively, followed by normal water for a further 2 days. POM‐1 (Tocris Bioscience, Bristol, UK) or PBS was injected intraperitoneally (i.p.) into indicated mice (20 mg per kg body weight per day) daily for 6 (for WT C57BL/6 mice) or 5 (for *Rag1* ^−*/*−^ mice) consecutive days from Day 2 after the beginning of DSS treatment. 1 μg of recombinant IL‐22 (PeproTech) or PBS was injected i.p. into indicated *Rag1* ^−*/*−^ mice daily for 7 consecutive days from the beginning of the experiments. Throughout the experimental timeline, mice were weighed and scored daily for a disease activity index (DAI) score to monitor colitis progression and pathology. The DAI was scored additively using the following parameters: body weight: 0 (no or <1% weight loss compared to Day 0 body weight), 1 (1–5% weight loss), 2 (5–10% weight loss), 3 (10–20% weight loss), and 4 (>20% weight loss); bleeding: 0 (no bleeding), 1 (blood present in/on feces), 2 (visible blood in rectum), and 4 (visible blood on fur); stool consistency: 0 (well formed/normal stool), 1 (pasty/semi‐formed stool), 2 (pasty stool with some blood), 3 (diarrhea that does not adhere to anus), and 4 (diarrhea that does adhere to anus); and general appearance: 0 (normal), 1 (piloerection only), 2 (piloerection and lethargy), and 4 (motionless, sickly, sunken‐eyed and ataxic). Mice were immediately culled if body weight loss was <25%, or the total colitis score was 12 or higher, or if their general appearance score was 4.

### Histology

Colon samples were fixed with 10% neutral buffered formalin solution (Sigma, Dorset, UK), embedded in paraffin, and 5 μm sections were used for staining with hematoxylin and eosin (H&E). Sections were photographed using a digital camera and white balance adjustment of entire images was done using Photoshop.

### Isolation of intestinal lamina propria cells

Small and large intestinal lamina propria (LP) cells were isolated as described previously.^22^ In brief, mice were culled and their small and large intestines were removed. After removing any remaining fatty and mesenteric tissues, samples were cut open longitudinally and any contents removed, washed with HBSS buffer containing 2% FCS, and then cut into 0.5 cm pieces. Intestines were shaken at 37°C for 15 min in HBSS and washed twice. Intestines were then transferred into gentleMACS C tubes (Miltenyi) digested in RPMI 1640 medium containing 10% FCS, 1.25 mg mL^−1^ collagenase IV (Roche) and 30 μg mL^−1^ DNase‐I (Roche) by shaking at 37°C for 30 min. Digested tissues were homogenized by gentleMACS disassociator running the programme m_intestine_01 and mashed through a 40‐μm cell strainer and flushed through HBSS containing 2% FCS. After centrifugation, cells were resuspended in complete RPMI 1640 medium for counting, staining, culture and/or sorting.

### *In vitro* cell culture

Intestinal LPLs or spleen cells were isolated from *Rag1* ^−*/*−^ mice and cultured in completed RPMI 1640 medium supplemented with 10% FBS, 2‐Mercaptoethanol (50 μmol L^−1^, Gibco, Waltham, MA, USA), l‐glutamine (2 mmol L^−1^, Gibco) and antibiotics (Penicillin and Streptomycin, 100 U mL^−1^, Gibco). When indicated, recombinant IL‐23 (20 ng mL^−1^, eBioscience, San Diego, CA, USA), POM‐1 (2–50 μmol L^−1^, Tocris Bioscience), APCP (2–50 μmol L^−1^), Apyrase (10 μmol L^−1^, Sigma), ATPγS (5–10 μmol L^−1^, Sigma), and A2A agonist CGS 21680 (1–10 μmol L^−1^, Merck Chemicals, Darmstadt, Germany) and their respective vehicle control (i.e. DMSO or dH_2_O) or combination of these reagents were added into cell cultures. For detecting cytokines in supernatants, cells were cultured for overnight, and for intracellular staining of IL‐22, cells were stimulated for 4 h in the presence of GolgiPlug (BD Bioscience, San Jose, CA, USA). *In vitro* cell cultures were performed in triplicate and repeated two or more times.

### Cell staining and flow cytometry

For surface staining, cells were first stained with the Fixable Viability Dye eFluor^®^ 780 on ice for 30 min to exclude dead cells. After wash, cells were stained on ice for another 30 min with indicated Abs including anti‐mouse CD45 eFlour^®^ 450 (Clone 30‐F11), CD3e PE, CD11c PE, CD11b PE, B220 PE (clone RA3‐6B2), CD90.2 FITC (Clone 30‐H12), CD4 PerCP‐Cyanine5.5 or APC, CD39 PE‐Cy7 (clone Duha59), CD11b FITC and Ly‐6G (Gr‐1) APC (clone RB6‐8C5). For intracellular staining of IL‐22, cells were stimulated with IL‐23 (20 ng mL^−1^) for 4 h in the presence of GolgiPlug (BD Bioscience). After staining with surface markers, cells were fixed by the Foxp3/Transcription Factor Fix and Staining Buffer for 2 h or overnight and then stained with anti‐human/mouse IL‐22 APC (clone IL22JOP) and anti‐mouse ROR‐γt PerCP‐eFluor710 (clone B2D) in the Perm/Wash Buffer on ice for 1 h. All antibodies were purchased from eBioscience or Biolegend. Flow cytometry was performed on the BD LSR Fortessa (BD Bioscience) and analyzed by FlowJo software (Tree Star).

### Enzyme‐linked immunosorbent assay (ELISA)

For detection of IL‐22 levels in supernatants of cell cultures, ELISA Ready‐SET‐Go!^®^ kits for mouse IL‐22 (eBioscience) were used according to the manufacturer’s instructions.

### Gene expression analysis of human colon biopsies

Gene transcription levels were measured by transcription microarray in a study by Vanhove *et al*.^27^ on human colonic mucosal biopsies with active UC (*n* = 74), inactive UC (*n* = 23) or controls (*n* = 11). Raw microarray data were retrieved from the Gene Expression Omnibus dataset GSE59071.^27^ After normalization, we detected two outliers in the active UC samples, which did not at all relate to any other samples and showed other quality shortcomings, and thus we removed those two samples prior to further analysis. Gene expression data were transformed into Z‐score values for presentation with comparisons by a nonparametric Kruskal–Wallis test with *post hoc* Dunn's multiple comparisons test. Analysis of the dataset GSE11223 was described previously.^34^ Gene expression data were transformed into Z‐score values by genes across samples for presentation. The list of human Th17‐ or ILC3‐related genes and IL‐22 signaling (responsive) genes were retrieved from previous reports.^29^, ^30^, ^31^, ^32^

### Statistics

Statistical significance between two groups was examined by a Student's *t*‐test, while a one‐way or two‐way analysis of variance (ANOVA) with *post hoc* Bonferroni's multiple comparisons test were used to evaluate multiple groups. Parametric or nonparametric tests were chosen based on the normality and variance of data distribution. Correlation analysis was calculated by Pearson's correlation coefficient (*r*). Statistical work was performed using the Prism 6 software (GraphPad) and *P *<* *0.05 was considered statistically significant.

## Conflict of Interest

The authors declare no conflict of interest.
