# Supplementary material for: Purine metabolism controls innate lymphoid cell function and protects against intestinal injury
Source: Immunol Cell Biol. 2018 Jul 10;96(10):1049–59. doi: 10.1111/imcb.12167 (PMC6248310; doi:10.1111/imcb.12167)
Supplement: Supplementary file 1 [file IMCB-96-1049-s001.pdf]

Supplementary Materials

# **Purine metabolism controls innate lymphoid cell function and protects against intestinal injury**

Running title: Purine metabolism in ILC3 and intestinal injury

Siobhan Crittenden[1*], Ashleigh Cheyne[1*], Alexander Adams[2*], Thorsten Forster[3*], Calum T. Robb[1], Jennifer Felton[1], Gwo-Tzer Ho[1], Dominik Ruckerl[4], Adriano G. Rossi[1], Stephen M. Anderton[1], Peter Ghazal[3,5], Jack Satsangi[2], Sarah E. Howie[1] and Chengcan Yao[1]

*These authors contributed equally to this work.

*Corresponding author. E-mail: Chengcan.Yao@ed.ac.uk

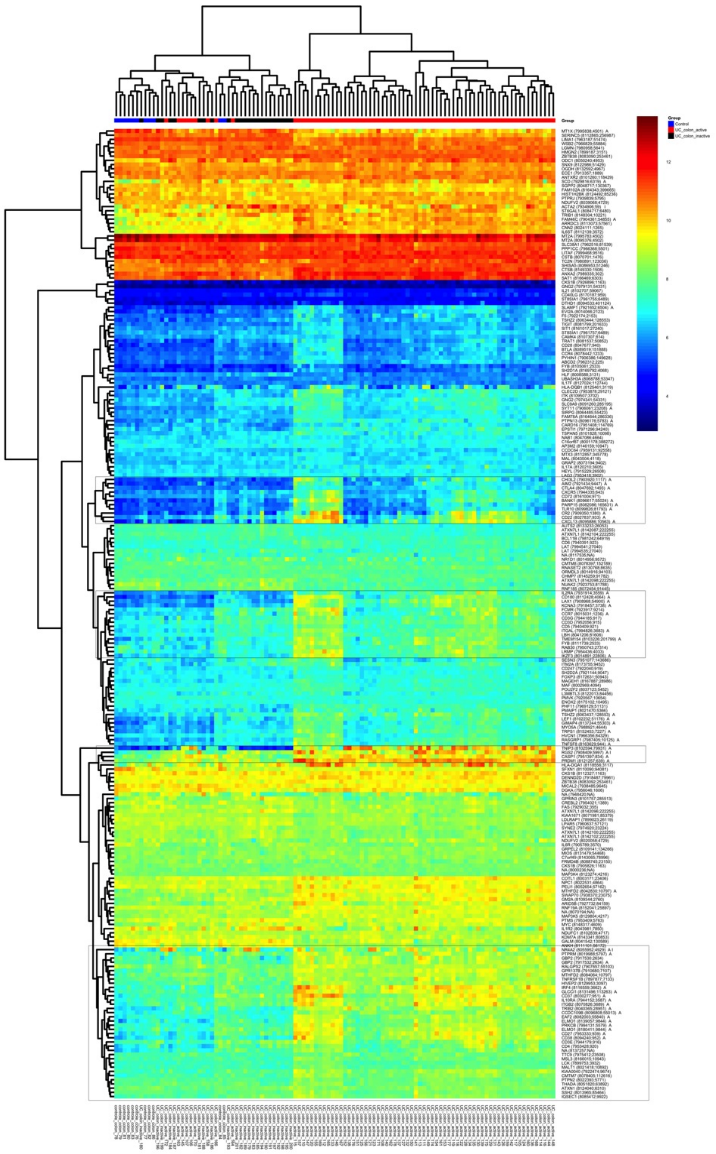

**Supplementary Figure 1. Expression of Th17 genes in colon biopsies of patients with UC.** Heat maps of expression of Th17 genes (33) in human colonic mucosal biopsies from patients with active UC (n=72) or inactive UC (n=23) and control colonic mucosal biopsies from healthy individuals (HC, n=11). Genes in rows and samples in columns, study group of sample is indicated by top colour bar (red=active ulcerative colitis). Cell colours reflect $log_2$ gene expression level from low (blue) to high (red). Dendrogram indicated the similarity of genes (or samples) based on a hierarchical clustering algorithm applied to the Euclidean distances between gene or sample expression profiles. Boxes outline notable gene clusters with correlated expression across samples. Microarray gene expression data were retrieved from the Gene Expression Omnibus dataset GSE59071[27].

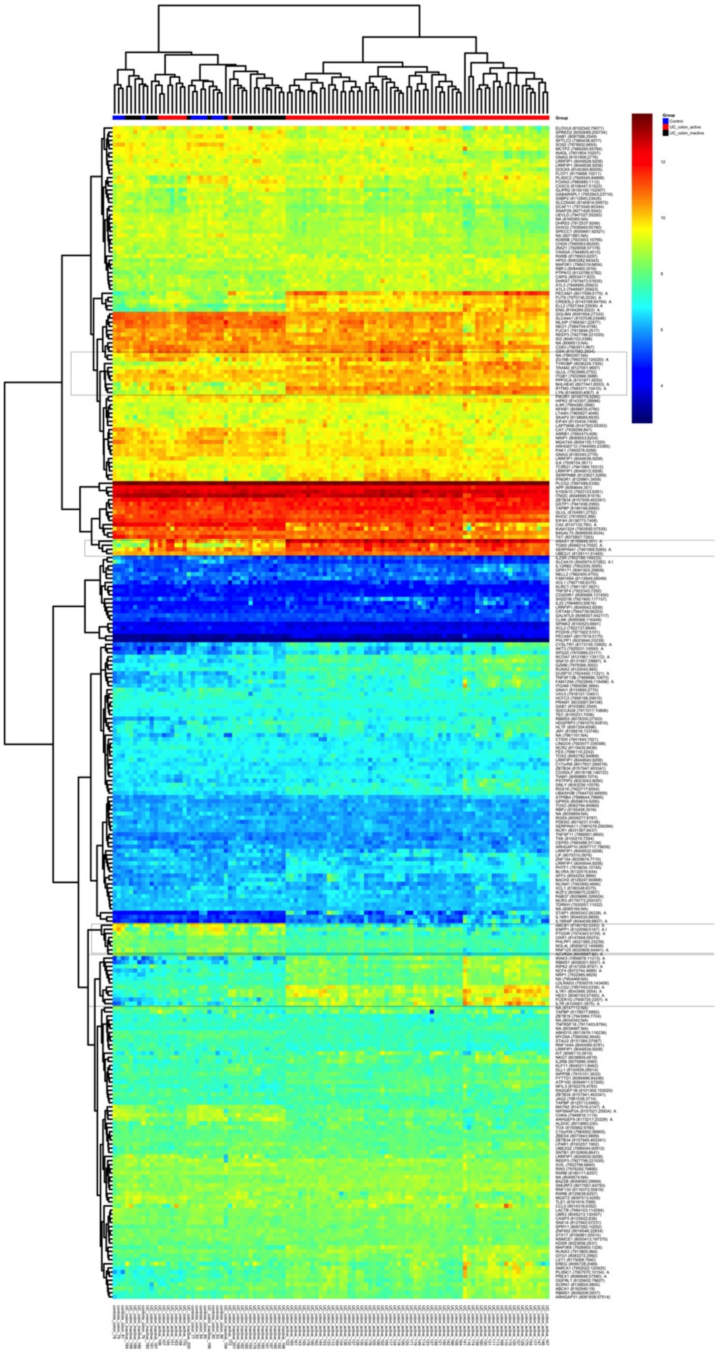

**Supplementary Figure 2. Expression of ILC3 genes in colon biopsies of patients with UC.** Heat maps of expression of ILC3 genes (32-34) in human colonic mucosal biopsies from patients with active UC (n=72) or inactive UC (n=23) and control colonic mucosal biopsies from healthy individuals (HC, n=11). Genes in rows and samples in columns, study group of sample is indicated by top colour bar (red=active ulcerative colitis). Cell colours reflect $\log_2$ gene expression level from low (blue) to high (red). Dendrogram indicated the similarity of genes (or samples) based on a hierarchical clustering algorithm applied to the Euclidean distances between gene or sample expression profiles. Boxes outline notable gene clusters with correlated expression across samples. Microarray gene expression data were retrieved from the Gene Expression Omnibus dataset GSE59071[27].

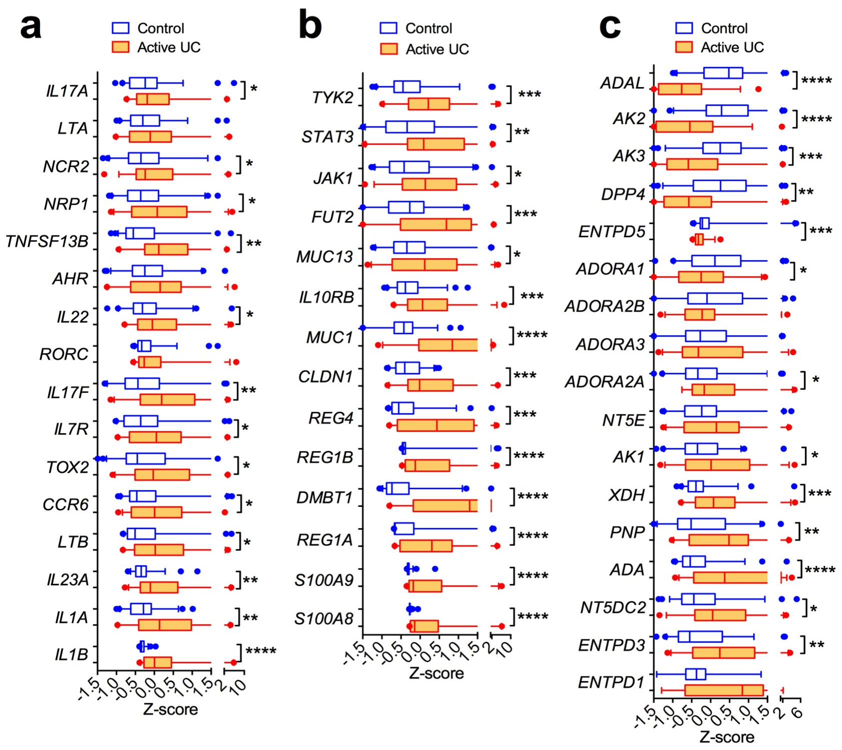


**Supplementary Figure 3. Gene expression in colon biopsies of patients with UC in another independent cohort.** Expression of selected genes related to human ILC3/Th17 **(a),** IL-22 signaling pathway **(b)** and purine metabolic pathway **(c)** in colonic mucosal biopsies from patients with active UC (n=32) or controls (n=56). Microarray gene expression data were retrieved from the Gene Expression Omnibus dataset GSE11223[34]. The active UC group was combined by colon samples without treatment and with treatment but non-responder. The z-score transformed values (by genes across samples) of microarray gene expression data were shown box and whiskers with 5-95 percentile. **P* < 0.05; ***P* < 0.01; ****P* < 0.001; *****P* < 0.0001 by Nonparametric Mann Whitney test.

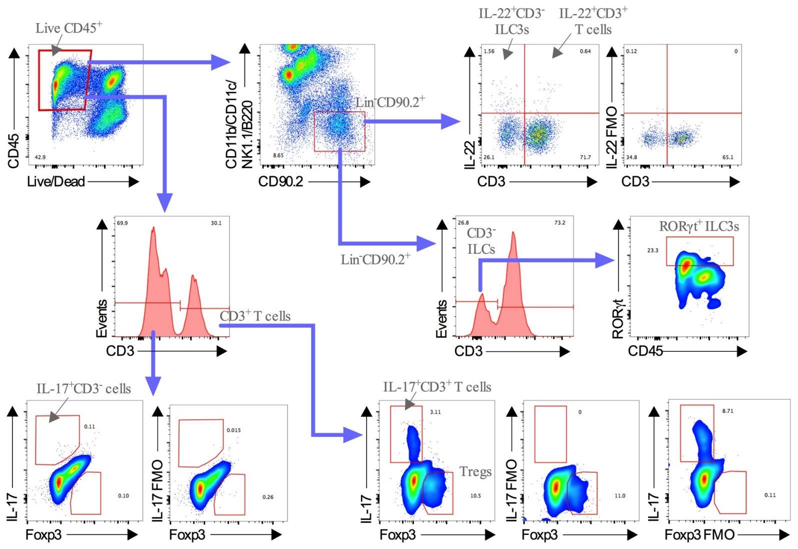


**Supplementary Figure 4.** Gating strategy for analyzing ILC3, Th17 and Treg cells in colon lamina propria. FMO, fluorescence minus one.

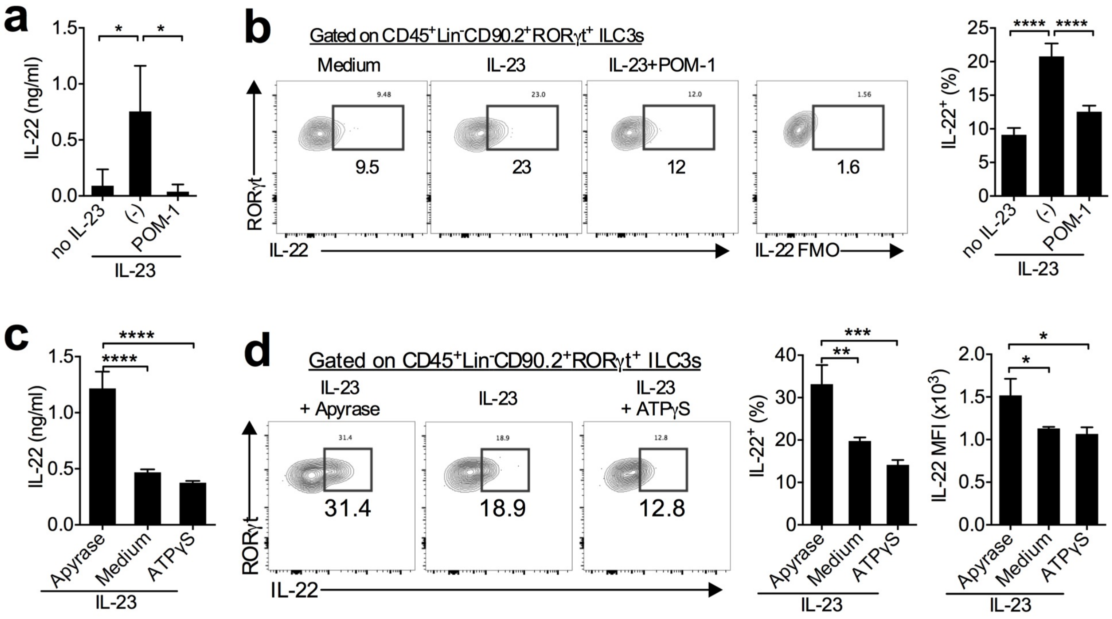


**Supplementary Figure 5. Regulation of IL-22 production from splenic ILC3s by NTPDases and eATP.** (**A,B**) Concentrations of IL-22 in supernatants (**a**) and percentages of IL-22$^+$ cells (**b**) by ILC3s isolated from spleens of *Rag1*$^{-/-}$ mice and then cultured with IL-23 plus POM-1 overnight (**a**) or for 4 h (**b**). (**c,d**) Concentrations of IL-22 in supernatants (**c**) and percentages of IL-22$^+$ cells (**d**) by ILC3s isolated from spleens of *Rag1*$^{-/-}$ mice and then cultured with IL-23 plus Apyrase or ATPγS overnight (**c**) or for 4 h (**d**). Data shown mean ± S.D. are representative two or more independent experiments. *$P < 0.05$, **$P < 0.01$, ***$P < 0.001$; ****$P < 0.0001$ by one-way ANOVA with post-hoc Bonferroni's multiple comparisons test.

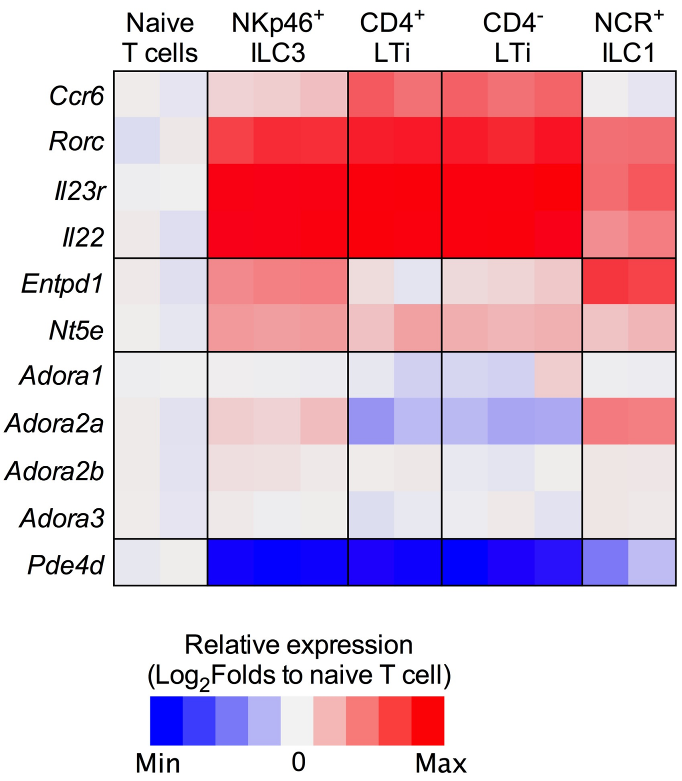


**Supplementary Figure 6. Gene expression of CD39 and adenosine receptors by mouse ILC3s.** Expression of *Entpd1*, *Nt5e* and genes encoding adenosine receptors (i.e. *Adora1,2a,2b,3*) as well as ILC3 signature genes in sorted LN TCR$^+$CD4$^+$CD8$^-$CD25$^-$CD62L$^{hi}$CD44$^{lo}$ Naïve T cells, siLP CD45$^+$CD3$^-$CD19$^-$ROR$\gamma$t$^{hi}$NKp46$^+$ ILC3s (NKp46$^+$ ILC3s), siLP CD45$^+$CD3$^-$CD19$^-$ROR$\gamma$t$^+$NKp46$^-$CD4$^+$ Lymphoid tissue inducer cells (CD4$^+$ LTis), siLP CD45$^+$CD3$^-$CD19$^-$ROR$\gamma$t$^+$NKp46$^-$CD4$^-$ Lymphoid tissue inducer cells (CD4$^-$ LTis), and siLP CD45$^+$CD3$^-$CD19$^-$ROR$\gamma$t$^-$NKp46$^+$CD127$^+$ NK1.1$^+$ ILC1s (NCR$^+$ ILC1s). Naïve T cells and ILC1s serve as negative and positive controls for *Entpd1* and *Nt5e* genes. Original microarray data were retrieved from the Gene Expression Omnibus dataset GSE37448[29].
